# Supplementary material for: Wing wettability gradient in a damselfly Lestes sponsa (Odonata: Lestidae) reflects the submergence behaviour during underwater oviposition
Source: R Soc Open Sci. 2020 Dec 16;7(12):201258. doi: 10.1098/rsos.201258 (PMC7813233; doi:10.1098/rsos.201258)
Supplement: Supplementary material 1 [file rsos201258supp1.docx]

**Supplementary material 1.** Post-hoc multiple comparisons of advancing and receding contact angles and contact angle hysteresis among individual wing parts (distal, middle, proximal).

|  | **contrast** | **estimate** | **SE** | **df** | **Z ratio** | **p-value** |
| --- | --- | --- | --- | --- | --- | --- |
| **Advancing CA** |  |  |  |  |  |  |
|  | proximal–middle | 0.099 | 0.0047 | inf | 21.335 | < 0.001 |
|  | proximal–distal | 0.355 | 0.0088 | inf | 40.341 | < 0.001 |
|  | middle–distal | 0.256 | 0.0072 | inf | 35.441 | < 0.001 |
| **Receding CA** |  |  |  |  |  |  |
|  | proximal–middle | 0.186 | 0.0112 | inf | 16.594 | < 0.001 |
|  | proximal–distal | 0.612 | 0.0303 | inf | 20.186 | < 0.001 |
|  | middle–distal | 0.426 | 0.0232 | inf | 18.347 | < 0.001 |
| **Hysteresis** |  |  |  |  |  |  |
|  | proximal–middle | -0.239 | 0.0393 | inf | -6.066 | < 0.001 |
|  | proximal–distal | -0.384 | 0.0559 | inf | -6.869 | < 0.001 |
|  | middle–distal | -0.146 | 0.0430 | inf | -3.387 | 0.002 |
