# Supplementary material for: Wing wettability gradient in a damselfly Lestes sponsa (Odonata: Lestidae) reflects the submergence behaviour during underwater oviposition
Source: R Soc Open Sci. 2020 Dec 16;7(12):201258. doi: 10.1098/rsos.201258 (PMC7813233; doi:10.1098/rsos.201258)
Supplement: Supplementary material 2 [file rsos201258supp2.docx]

**Supplementary material 2**

SEM photographs of the nanostructures of the wing membrane of *Lestes sponsa*. Wax rods of the different level of damage were observed:

a) intact wax rods

















b) wax rods slightly joined together indicating slightly more damaged structure

















c) wax structures quite intact but dirty

















d) wax rods strongly joined together, forming a damaged, network-like structure

















e) wax rods both damaged and dirty

















f) wax layer damaged or irretrievably lost
